# Peripheral Blood Mononuclear Cell Proteome Changes in Patients with Myelodysplastic Syndrome

**DOI:** 10.1155/2015/872983

**Published:** 2015-04-16

**Authors:** Klara Pecankova, Pavel Majek, Jaroslav Cermak, Jan E. Dyr

**Affiliations:** Institute of Hematology and Blood Transfusion, U Nemocnice 1, 128 20 Prague 2, Czech Republic

## Abstract

Our aim was to search for proteome changes in peripheral blood mononuclear cells (PBMCs) of MDS patients with refractory cytopenia with multilineage dysplasia. PBMCs were isolated from a total of 12 blood samples using a Histopaque-1077 solution. The proteins were fractioned, separated by 2D SDS-PAGE (pI 4–7), and double-stained. The proteomes were compared and statistically processed with Progenesis SameSpots; then proteins were identified by nano-LC-MS/MS. Protein functional association and expression profiles were analyzed using the EnrichNet application and Progenesis SameSpots hierarchical clustering software, respectively. By comparing the cytosolic, membrane, and nuclear fractions of the two groups, 178 significantly (*P* < 0.05, ANOVA) differing spots were found, corresponding to 139 unique proteins. Data mining of the Reactome and KEGG databases using EnrichNet highlighted the possible involvement of the identified protein alterations in apoptosis, proteasome protein degradation, heat shock protein action, and signal transduction. Western blot analysis revealed underexpression of vinculin and advanced fragmentation of fermitin-3 in MDS patients. To the best of our knowledge, this is the first time that proteome changes have been identified in the mononuclear cells of MDS patients. Vinculin and fermitin-3, the proteins involved in cell adhesion and integrin signaling, have been shown to be dysregulated in MDS.

## 1. Introduction

MDS encompasses a diverse range of oncohematological diseases affecting hematopoietic stem cells and their hematopoietic microenvironment [1]. MDS is characterized by dysplastic ineffective hematopoiesis with the apoptosis of hematopoietic cells in the bone marrow and by subsequent cytopenias in the blood. It occurs in particularly elder people with incidence of 20–50 patients in 100,000 inhabitants [2]. There are several groups of MDS patients according to the WHO classification based on bone marrow and peripheral blood findings, cytogenetics, and other factors [3]. Prognostically MDS subgroups can be also stratified into low-risk and high-risk subgroups; high-risk subgroups are characterized by poor survival outcome and higher rate of progression toward acute myeloid leukemia. Refractory cytopenia with multilineage dysplasia (RCMD) is a subgroup of myelodysplastic syndrome (MDS). According to the revised WHO (World Health Organization) classification of MDS, RCMD is defined by the presence of cytopenias in peripheral blood and dysplastic changes present in 10% or more of the cells in two or more myeloid lineages in the bone marrow (with approximately 15% ringed sideroblasts) [3].

In recent years, fundamental knowledge in MDS pathophysiology based on DNA alterations has been and is still being complemented by other “*omics*” disciplines, in particular by proteomics, with the proteomes of plasma (of different MDS subgroups such as refractory anemia and refractory anemia with ringed sideroblasts (RA and RARS) [4], RCMD [5], refractory anemia with excess of blasts subtype 1 (RAEB-1) [6], and refractory anemia with excess of blasts subtype 2 (RAEB-2) [7]), serum [8, 9], platelets [10], and neutrophils [11] of MDS patients having been investigated. Despite research efforts the pathogenesis and exact mechanisms of MDS development still remain unclear, as there are many factors involved. For example, factors involved in the development of MDS (cytogenetic abnormalities [12], gene mutations [13–15], epigenetic alterations [16], etc.) can contribute to the dysregulation of various processes in the immune system [17], a portion of which are by mononuclear blood cells (B-lymphocytes, T-lymphocytes, dendritic cells, and monocytes). Mononuclear cells represent a heterogeneous population; nevertheless, due to the rapid and simple methods of their isolation, they are believed to be a promising and interesting material to search for biomarkers [18–20]. As very little is known about the role of mononuclear cells and their protein alterations in MDS the goal of this work, our aim, has been to describe the changes in the proteome of mononuclear blood cells of MDS patients and to discuss the involvement of these changes in MDS pathophysiology.

## 2. Methods

In this pilot study, a total of 12 samples (RCMD *n* = 6, control *n* = 6) have been investigated. The diagnosis of RCMD was established according to the WHO classification criteria [21]. The median age of RCMD patients was 67; the patient group included 4 females (67%). The median age of sex-matched healthy control donors was 28. Patient characteristics are summarized in Table 1. All of the individuals tested agreed to participate in the study on the basis of an informed consent. All samples were obtained and analyzed in accordance with the Ethical Committee regulations of the Institute of Hematology and Blood Transfusion.

Blood samples were collected by venipuncture into EDTA-coated tubes. Peripheral blood mononuclear cells were isolated from 9 mL of whole blood using Histopaque-1077 (Sigma-Aldrich, Prague, Czech Republic) according to manufacturer instructions.

Protein fractionation was performed using a ProteoExtract Subcellular Proteome Extraction Kit (Merck Millipore, Darmstadt, Germany) according to manufacturer instructions to enrich the proteins according to their subcellular localization; four different subproteomes were obtained (cytosolic, membrane and membrane organelle, nuclear, and cytoskeletal). Enriched proteins were precipitated with the addition of four volumes of acetone, incubated for 60 min at −20°C, and then centrifuged at 15,000 ×g for 10 min. Protein concentration in all samples was determined using a Micro-BCA Protein Assay Kit (Thermo Fischer Scientific, Waltham, MA, USA). Protein sample concentrations of each subproteome were adjusted to the same level.

Isoelectric focusing was performed (IPG strips pI 4–7, 7.7 cm) followed by SDS-PAGE (8 × 10 cm, 10% resolving gel, 3.75% stacking gel, and 30 mA/gel), as described in detail in previous publications [4–6, 22]. Briefly, 40 *μ*g of cytosolic, 50 *μ*g of membrane and membrane organelle, and 40 *μ*g of nuclear proteins were used for an IPG strip. The proteins of the cytoskeletal subproteome were not analyzed due to insufficient protein yield.

The gels were double-stained according to the improved fast-staining protocol [23], combining imidazole-zinc reverse and Coomassie dye-based staining. Imidazole-zinc reverse staining was used to detect as many spots as possible, followed by Coomassie dye-based staining to enable maximal spot detection and quantification. After each staining step, the gels were digitized and processed with Progenesis SameSpots software (Nonlinear Dynamics, Newcastle upon Tyne, UK) that computed the fold and *P* values of all spots using one-way ANOVA analysis. Protein spots that differed significantly (*P* < 0.05) were submitted for protein identification by tandem mass spectrometry (HCT ultra ion-trap mass spectrometer with nanoelectrospray ionization; Bruker Daltonics, Bremen, Germany) coupled to a nano-LC system (UltiMate 3000; Dionex, Sunnyvale, CA, USA); this procedure has been described in detail previously [4–6, 22]. Mascot (Matrix Science, London, UK) was used for database searching (Swiss-Prot). Two unique peptides (with higher Mascot scores than the minimum for identification, *P* < 0.05) were necessary to successfully identify a protein. Exceptions were given to proteins with a molecular weight of 15 kDa or less and to proteins with more than 3 additional unique peptides identified by error tolerant search.

To analyze the functional associations between identified proteins and cellular pathways, the protein list was processed with the on-line EnrichNet application [24] using KEGG [25, 26] and Reactome [27, 28] databases. The significance of overlap between protein sets was evaluated using a combination of one-side Fisher's exact test (*q* < 0.05) and network similarity scores (XD-scores). The threshold values were estimated via EnrichNet with a regression fit equivalent to a Fisher *q* value of 0.05 and an upper boundary of 95% confidence for linear fitting.

Dendrogram analysis was performed using Progenesis SameSpots software to reveal closely related proteins. The dendrogram is a visual representation of spot correlation data (with correlation analysis performed on log-normalized spot expression levels). Spots were clustered according to their closest correlation.

Western blot analysis was performed for vinculin and fermitin-3 proteins. Equal protein amounts of all (patient or donor) samples of appropriate protein fractions were pooled and 1 *μ*g or 0.75 *μ*g of total protein amounts were used for 1D western blot analyses for vinculin or fermitin-3, respectively. Briefly, following SDS-PAGE (10% resolving gel) proteins were transferred to a PVDF membrane (10 V constant voltage for 60 min) using an Owl HEP-1 Semi Dry Electroblotting System (Thermo Scientific, Waltham, MA, USA). Membranes were then incubated with a blocking buffer (3% BSA in PBS) at 30°C for 60 min and incubated with primary antibodies, anti-vinculin antibody (V9131; Sigma-Aldrich, Praque, Czech Republic) (1 : 200 dilution) or anti-kindlin-3 antibody (SAB4200013; Sigma-Aldrich, Prague, Czech Republic) (1 : 340 dilution). Then the membranes were incubated with secondary antibodies, rabbit anti-mouse IgG antibody conjugated with peroxidase (for vinculin detection, 1 : 80,000 dilution) (A9044; Sigma-Aldrich, Prague, Czech Republic) or goat anti-rabbit IgG antibody conjugated with peroxidase (for kindlin-3 detection, 1 : 120,000 dilution) (A0545; Sigma-Aldrich, Prague, Czech Republic). Visualization was performed using a chemiluminescent substrate (SuperSignal West Pico; Thermo Scientific, Waltham, MA, USA) and CL-XPosure Film (Thermo Scientific, Waltham, MA, USA).

## 3. Results and Discussion

**Figure 1:**
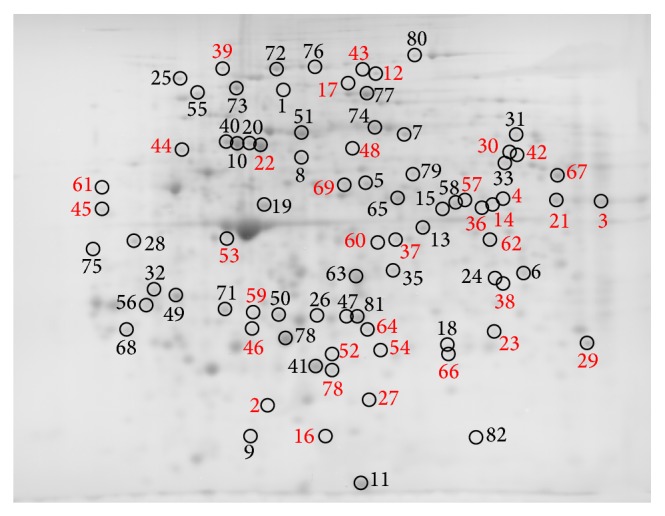
Positions of cytosolic subproteome spots significantly differing in normalized volumes, only in gels stained by imidazole-zinc (red numbers), only in Coomassie stained gels (blue numbers), and in both stains (black numbers). Brightness and contrast of the gel image were adjusted for more clear illustration.

**Figure 2 fig2:**
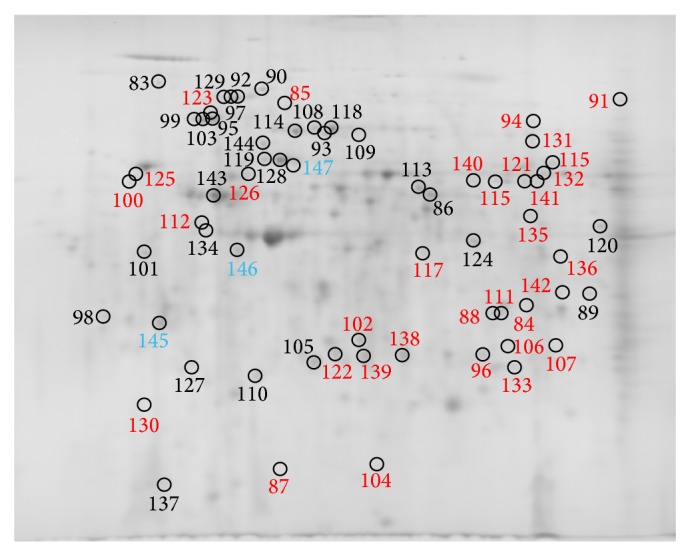
Positions of membrane and membrane organelle subproteome spots significantly differing in normalized volumes, only in gels stained by imidazole-zinc (red numbers), only in Coomassie stained gels (blue numbers), and in both stains (black numbers). Brightness and contrast of the gel image were adjusted for more clear illustration.

**Figure 3:**
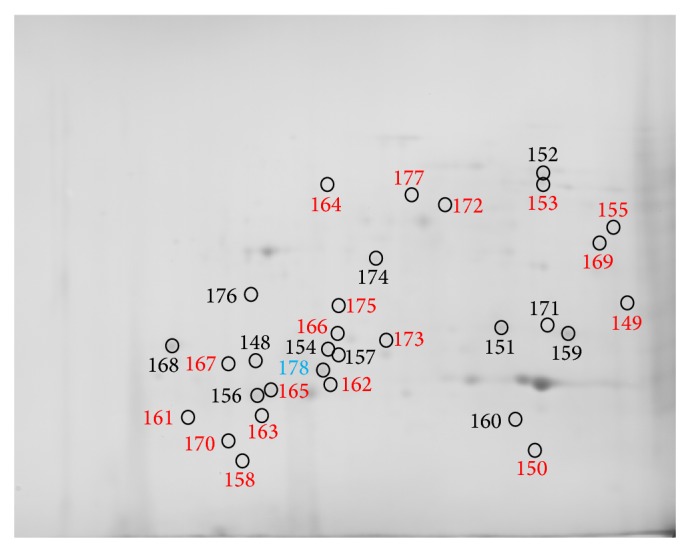
Positions of nuclear subproteome spots significantly differing in normalized volumes, only in gels stained by imidazole-zinc (red numbers), only in Coomassie stained gels (blue numbers), and in both stains (black numbers). Brightness and contrast of the gel image were adjusted for more clear illustration.

Comparing the PBMC subproteomes of RCMD patients (*n* = 6) with healthy volunteer control group subproteomes (*n* = 6), we found 178 spots that differed significantly in their normalized volumes (*P* < 0.05). Figures 1 [Fig fig2]–3 indicate the positions of significantly differing spots of the cytosolic, membrane and membrane organelle, and nuclear subproteomes, respectively. The spots are marked considering the gel staining.

Proteins of the spots detected were submitted to protein identification by mass spectrometry, and 139 unique proteins were identified. The list of all spots, including ANOVA *P* values, their multiplication (fold value), protein identification with the number of identified peptides (unique peptides above the identity threshold score), and protein accession number (Swiss-Prot), is summarized in Table 2.

We compared these summarized results with our previous research that investigated the plasma proteomes of patients with different MDS subgroups [4–7]. It is known that PBMCs can secrete proteins into the plasma [29, 30]; therefore, we searched for changes in plasma proteomes that could be related to PBMCs. Nevertheless, none of the proteins identified in this study corresponded to any proteins identified in our previous proteomic studies of the plasma. This observation strongly indicates that the alterations observed in plasma proteins (possibly secreted by PBMCs) are caused by posttranslational modifications of such proteins instead of the changes in their level. For example, we have shown in our previous plasma proteome MDS studies [4–7] that inter-alpha-trypsin inhibitor heavy chain H4 (ITIH4) was extensively fragmented and the changes observed (for ITIH4) were related to these fragments; similar results were observed for complement proteins. However, it should be taken into account that PBMCs are not the only source of these plasma proteins.

In order to estimate the possible involvement of the identified proteins in the PBMC cellular and metabolic pathways and thus reveal the processes influenced by RCMD pathogenesis, we processed the protein dataset in EnrichNet. Enrichment analysis using KEGG and Reactome databases revealed implications of the identified proteins in several cellular pathways (Tables 3 and 4). XD-scores were considered to be more than 0.78 and 1.0 threshold values, as estimated by the application for the KEGG and Reactome databases, respectively.

Dendrogram analysis was performed using Progenesis SameSpots, which grouped spots by their expression profiles using an automatic correlation analysis and hierarchical clustering. We chose the top ten spot expression profile groups (with distance parameters less than 0.5) with almost identical expression profiles. Thus, each group contained spots with similar expression profiles, suggesting that these spots may be coregulated, colocalized, or by another way coaffected. The list of the expression profile groups (denoted as A, B, etc.), including the spot number, protein identification, and its accession number, is presented in Tables 5–7. Proteins in the F1 groups (cytosolic subproteome) were associated with the cytoskeleton, microtubule metabolism, cellular homeostasis maintained by heat shock proteins (HSPs), and proteasome. Proteins in the F2 groups (membrane and membrane organelle subproteome) were associated with the mitochondria and apoptosis. The protein identified in most cases in the F3 groups (nuclear subproteome) was Filamin-A, which is released from the apoptotic nucleus [31]. Other proteins in these groups were mainly actin and actin-binding proteins. Thus, the actual associations corresponded to the subproteomes as anticipated. Due to thematic similarity of the protein groups obtained via EnrichNet and dendrogram analysis, we discuss the results in parallel. Further in relation to MDS or other hematological malignancies, we summarize the most interesting protein groups that could be affected in connection with pathophysiological processes.

PBMCs are metabolically active cells (carbohydrate metabolism, cellular respiration) and as cells of the immune system are involved in antigen processing and presentation. Our KEGG results analysis (Table 3) highlighted the relationships of the identified proteins to infectious agents (*E. coli*). This observation is not surprising, as it is known that the immune system in MDS is dysregulated and MDS patients tend to be especially vulnerable to infections [17, 32]. Therefore, this observation is most likely related to the manifestation of secondary complications, rather than MDS itself.

T-complex protein 1 (TCP-1) subunits (four of eight identified, Table 2) with a degree of functional autonomy [33] are a part of the TRiC cytosolic chaperone (TCP-1 ring complex), which acts in tubulin biosynthesis (Table 4). This chaperone was originally thought to fold only cytoskeletal proteins but now is known to have a more general role in protein folding in eukaryotic cytosol [34]. TRiC also assists in the formation of BBSome, a part of the primary cilium, nonmotile microtubule-based sensory organelle transporting signals within the cell [35]. Primary cilium has not been under closer analysis until in the last decade, and many questions surrounding it are still unanswered. For example, it is not entirely known, whether the primary cilium is present in PBMCs [36–39]. There is evidence that components which contribute to the assembly of the primary cilium are expressed by PBMCs [36]. Therefore, there is a possibility that TCP subunits are part of the machinery of primary cilium formation or function, as a chaperone not only of cytoskeletal proteins (see Table 5, Groups F1A, F1B). Changes in TCP subunits could cause changes or even errors in BBSome formation [40], thus an effect on signaling within the cell. In last decade, the function of primary cilium in several cancers has been described, but its role in hematological malignancies has not yet been unraveled. There is also the possibility that a change in TCP subunits could affect the proper folding of proteins involved in hematopoiesis and its regulation and therefore may contribute to MDS pathogenesis.

Apoptosis, an important phenomenon in MDS, in a highly regulated manner removes the excess or potentially dangerous cells from the organism. The apoptotic process relies heavily on the cleavage of proteins/proteolysis. Any proteolytic pathway involved in cell death regulation must be precise; therefore, a highly regulated proteasome pathway is a good candidate for the regulation of protein composition during apoptosis [41]. Evidence of cross talk between the apoptotic pathways, HSPs, and proteasome system exists [42]; the relation of these processes is also suggested in our dendrogram analysis results (see Table 5, Group F1C). It is difficult to define a clear role or the involvement of the proteasome system in apoptosis, because in some systems proteasome activity induces apoptosis while in others it does not [42]. It is possible that increased proteasome activity causes the suppression of apoptosis, and this could be one of the reasons for the transformation from MDS to AML [43]. In relation to programmed cell death, researchers have identified changes in the regulatory subunits of the 19S cap complex [44, 45]. We observed changes in several proteasomal proteins and in the non-ATPase regulatory subunits of the 26S complex (Table 5, Group F1C), a part of which is the 19S cap complex [46]. We also observed changes in several HSPs, including HSP90*α* (identified in spot 51), whose overexpression has already been linked to apoptosis and MDS [47]. HSPs assist in proper protein folding, as molecular chaperones. They are fundamental for cell life and death decisions, and their abnormal expression is linked to oncogenesis [48]. When a protein is misfolded, HSPs are induced and associate with the misfolded protein, trying to refold it. When this process fails, the protein is ubiquitinated and determined to be processed by the proteasome. In case there are not enough HSPs or the proteasome function is impaired, proteins tend to aggregate with HSPs, ubiquitin, and proteasome to an insoluble compartment and trigger apoptosis [42, 49, 50]. HSPs can act in apoptosis at three levels: (i) in upstream mitochondria by regulating signaling molecules [51] (see Table 5, Group F1C); (ii) at the mitochondrial level by controlling mitochondrial membrane permeabilization and thus the release of cytochrome c [52] (see Table 6); and (iii) downstream of the mitochondria by affecting apoptosome formation [53] (see Table 5, Group F1C). Their role in apoptosis is also controversial; HSP function in apoptosis may be impacted by posttranslational modifications and the interaction with cochaperones (e.g., the DnaJ-family proteins identified in spot 124) [54]. The overexpression of HSPs has been shown to block apoptosis; and on the other hand, the depletion of HSPs increases sensitivity to apoptotic stimuli [55]. We observed both a decrease and an increase in normalized volumes in the spots containing HSPs (Table 2). However, from 2D SDS-PAGE data it is not possible to claim whether a change is caused by protein expression alteration or by protein posttranslational modification. Therefore, there is the possibility that the HSPs identified are posttranslationally modified, differently expressed, or a combination of both. Changes in proteasomal proteins and HSPs could be involved in cells' decisions regarding the triggering of apoptosis. Because of the inconsistency in the roles of both the proteasome and HSPs in apoptosis, we can only speculate whether the changes are the cause of the apoptosis in MDS.

**Figure 4:**
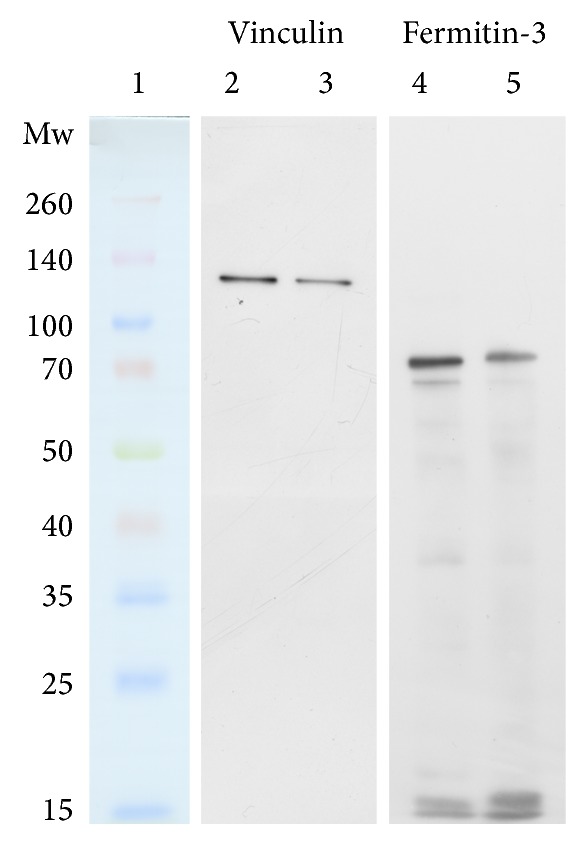
Western blot analysis of vinculin and fermitin-3 proteins; 1, molecular weight marker (kDa); 2 and 4, pooled control samples; 3 and 5, pooled MDS patient samples.

In order to provide further insight into the possible PMBCs alterations in MDS patients additional to those suggested by the 2D electrophoresis data, western blot analysis was performed for the two of identified proteins: fermitin-3 and vinculin. Both the proteins were uniquely identified in the corresponding spots (without coidentified proteins) and they are both involved in particular in cell adhesion and integrin signaling [56, 57]. While vinculin was identified in five different spots with both increasing and decreasing spot normalized volumes (which strongly indicates the presence of posttranslational modifications and the alterations of individual proteoforms), fermitin-3 was observed in one spot only. The results of western blot analysis are shown in Figure 4.

It is apparent that vinculin is underexpressed in PBMCs of MDS patients when compared to the healthy donor group. Therefore, while the prevalent vinculin form is decreased in MDS there is also a minority of posttranslationally modified forms altered as estimated from 2D electrophoresis and LC-MS/MS data. Vinculin is an actin-binding protein that is involved especially in cell adhesion dynamics and cell migration [56, 58]. Downregulation of vinculin expression could be possibly related to the immune system dysregulation in MDS as vinculin underexpression was described in the study by Kim et al. [56] investigating proteome changes in PBMCs of patients with atopic dermatitis, a chronic inflammatory skin disease. The presence of vinculin posttranslational modifications (as indicated in this work) was also observed in the proteomic study of PBMCs collected from amyotrophic lateral sclerosis (ALS) patients; significantly higher level of vinculin nitration was observed for sporadic ALS patients compared to healthy donors [59].

Western blot analysis of fermitin-3 showed the presence of several protein forms; in addition to the uncleaved protein, various fragments of fermitin-3 were observed. The uncleaved fermitin-3 band was observed with a lower intensity in MDS patients compared to the healthy controls; this observation may suggest fermitin-3 underexpression in MDS patients. However, the total intensity of the unaltered fermitin-3 together with its fragments estimated with ImageJ software [60] showed that there is no difference between MDS patients and the healthy controls. Therefore, while fermitin-3 expression did not seem to differ between the studied groups, it was shown that there was advanced fermitin-3 fragmentation in MDS patients. Fermitin-3 is a member of the family of focal adhesion proteins exclusively expressed in hematopoietic cells [61]. Its role in adhesion is essential for the function of blood cells including leukocytes [57, 62, 63]. Thus, higher rate of fermitin-3 fragmentation observed in this work could substantially influence PMBCs function in MDS. Moreover, fermitin-3 and vinculin are known to be colocalized to hematopoietic cell adhesion structure called podosome [64]. Therefore, since vinculin and fermitin-3 are both involved in cell adhesion and integrin signaling (and thus could play an important role in clinical applications) their changes observed in this work could initiate future research efforts.

## 4. Conclusion

In conclusion, we have compared the peripheral blood mononuclear cell proteome of myelodysplastic syndrome patients with refractory cytopenia with multilineage dysplasia against the proteome of healthy donors using two-dimensional electrophoresis combined with mass spectrometry. Through data mining of the Reactome and KEGG databases using EnrichNet, we highlighted the possible involvement of the identified protein alterations in apoptosis, protein degradation by proteasome, heat shock protein action, and signal transduction. Western blot analysis showed substantial changes in vinculin and fermitin-3, proteins involved in cell adhesion and integrin signaling. Vinculin was found to be underexpressed in MDS patients; advanced fragmentation of fermitin-3 was shown to take place in PBMCs of MDS patients.

To the best of our knowledge, our pilot study represents the first report on the proteome changes of peripheral blood mononuclear cells in myelodysplastic syndrome.

## Figures and Tables

**Table 1:** Patient characteristics.

Patient	1	2	3	4	5	6
Sex	F	F	F	F	M	F
Age	70	33	69	59	73	65
Hb [g/L]	119	83	91	92	117	104
RBC [10^12^/L]	3.15	2.47	2.33	2.67	4.29	2.87
WBC [10^9^/L]	2.20	2.81	5.50	4.15	5.10	2.89
PLT [10^9^/L]	184	291	262	211	50	284
NS	1.1	1.91	3.08	2.41	2.3	0.72
Blasts in PB [%]	0	0	0	0	0	0
Blasts in BM [%]	0.4	0.9	3.2	4.8	0.8	3.4
Karyotype	46, XX, inv(9)	46, XX	46, XX, del(5)(q13.2q34)	46, XX, del(5)(q31.q31)	46, XY	46, XX, 1qh+, 46, XX, 1qh+, del(5)(q31)
IPSS	0.5	0	0	0	0	0.5
IPSS-R	Low	Low	Low	Low	Very low	low
WPSS	Low	Low	Low	Low	Intermediate	intermediate
Transfusion [avg./month]	0.50	4.50	3.42	3.25	1.47	2.19

Hb: hemoglobin, RBC: red blood cells, WBC: white blood cells, PLT: platelets, NS: neutrophil segments, PB: peripheral blood, BM: bone marrow, IPSS (-R): International Prognostic Scoring System (-Revised), and WPSS: WHO Classification-Based Prognostic Scoring System.

**Table 2:** List of spots that differed significantly when RCMD patients were compared with a control group of healthy volunteers.

Spot	Protein	Uniprot AC	Peptides	*P*	Fold
1	Vinculin	P18206	5	0.0017	2.7

2	Tubulin alpha-1C chain; tubulin alpha-1B chain	Q9BQE3; P68363	4	0.00478	2.6
	Annexin A6	P08133	2		

3	Alpha-enolase	P06733	8	0.026	−2.5

4	Alpha-enolase	P06733	6	0.0345	−2.4

5	Cytosolic nonspecific dipeptidase	Q96KP4	14	0.00179	−2.3
	Tubulin beta chain	P07437	5		

6	Annexin A1	P04083	10	0.00387	−2.4

7	Leukotriene A-4 hydrolase	P09960	12	0.0000401	−2.1

8	T-complex protein 1 subunit theta	P50990	6	0.00287	−1.7

9	Actin, cytoplasmic 1; actin, cytoplasmic 2	P60709; P63261	3	0.0133	2.1

10	Plastin-2	P13796	17	0.000467	−3.3

11	Protein S100-A9	P06702	1	0.0259	−2.0

12	Neutral alpha-glucosidase AB	Q14697	3	0.012	−2.0

13	Leukocyte elastase inhibitor	P30740	8	0.0114	−2.0

14	Alpha-enolase	P06733	4	0.000782	−2.0
	Adenylosuccinate synthetase isozyme 2	P30520	5		
	Rab GDP dissociation inhibitor beta	P50395	3		
	C-terminal-binding protein 1	Q13363	2		

15	Annexin A7	P20073	3	0.00019	−2.0
	Adenosylhomocysteinase	P23526	3		
	Proliferation-associated protein 2G4	Q9UQ80	1		

16	Actin, cytoplasmic 1; actin, cytoplasmic 2	P60709; P63261	3	0.0247	1.9
	T-complex protein 1 subunit beta	P78371	2		

17	Vinculin	P18206	5	0.000267	1.9

18	Triosephosphate isomerase	P60174	5	0.000113	−1.7

19	Eukaryotic initiation factor 4A-I	P60842	6	0.000589	−1.9
	Heterogeneous nuclear ribonucleoprotein F	P52597	4		
	Heterogeneous nuclear ribonucleoprotein Q	O60506	4		
	T-complex protein 1 subunit theta	P50990	3		

20	Plastin-2	P13796	14	0.00156	−1.9

21	Alpha-enolase	P06733	7	0.00129	−1.9

22	Plastin-2	P13796	16	0.0363	−1.9

23	Proteasome subunit alpha type-6	P60900	4	0.000319	−1.8
	UPF0568 protein C14orf166	Q9Y224	4		
	Hypoxanthine-guanine phosphoribosyltransferase	P00492	2		

24	Alpha-enolase	P06733	6	0.0172	1.8

25	Ubiquitin carboxyl-terminal hydrolase 5	P45974	5	0.00228	−1.7

26	Proteasome activator complex subunit 1	Q06323	2	0.0017	1.7
	Coagulation factor XIII A chain	P00488	2		

27	Heat shock cognate 71 kDa protein	P11142	4	0.00034	−1.8
	Rho-related GTP-binding protein RhoC	P08134	2		

28	40S ribosomal protein SA	P08865	7	0.00126	−1.8

29	Triosephosphate isomerase	P60174	7	0.0217	−1.8

30	T-complex protein 1 subunit zeta	P40227	4	0.00972	−1.8

31	WD repeat-containing protein 1	O75083	9	0.0183	−1.8

32	Actin, cytoplasmic 1; actin, cytoplasmic 2	P60709; P63261	2	0.00152	1.7
	Heterogeneous nuclear ribonucleoprotein K	P61978	2		
	ADP-sugar pyrophosphatase	Q9UKK9	2		

33	Coronin-1A	P31146	2	0.00433	−1.7

34	26S proteasome non-ATPase regulatory subunit 14	O00487	2	0.000458	−1.7

35	m7GpppX diphosphatase	Q96C86	4	0.00316	−1.5
	Transaldolase	P37837	3		

36	Proliferation-associated protein 2G4	Q9UQ80	3	0.000667	−1.7
	Annexin A1	P04083	2		

37	Macrophage-capping protein	P40121	3	0.0279	−1.7

38	Talin-1	Q9Y490	5	0.00497	1.7

39	Heat shock 70 kDa protein 4	P34932	5	0.0282	−1.7

40	Plastin-2	P13796	17	0.0131	−1.6

41	Glutathione S-transferase P	P09211	6	0.000628	−1.8

42	Protein S100-A7	P31151	2	0.00218	−1.6

43	Talin-1	Q9Y490	4	0.00804	1.6

44	Serine/threonine-protein phosphatase 2A 65 kDa regulatory subunit A alpha isoform	P30153	7	0.00245	−1.6

45	Ribonuclease inhibitor	P13489	3	0.0247	−1.6

46	Actin, cytoplasmic 1; actin, cytoplasmic 2	P60709; P63261	3	0.00181	1.6

47	Tubulin beta-1 chain	Q9H4B7	2	0.0144	1.6

48	Vinculin	P18206	3	0.0104	1.6

49	Annexin A5	P08758	12	0.00129	−1.8

50	Proteasome activator complex subunit 2	Q9UL46	3	0.000338	−1.6

51	Annexin A6	P08133	20	0.000508	−2.6
	Heat shock 70 kDa protein 1A/1B	P08107	3		
	Heat shock 70 kDa protein 1-like	P34931	2		
	Heat shock protein HSP 90-alpha	P07900	2		

52	Actin, cytoplasmic 1; actin, cytoplasmic 2	P60709; P63261	2	0.000127	−1.6
	Glutathione S-transferase P	P09211	3		

53	Actin, cytoplasmic 1; actin, cytoplasmic 2	P60709; P63261	2	0.0352	1.5

54	Triosephosphate isomerase	P60174	2	0.00181	−1.5

55	Ras GTPase-activating-like protein IQGAP1	P46940	13	0.0219	−1.7

56	Actin, cytoplasmic 1; Actin, cytoplasmic 2	P60709; P63261	2	0.019	1.5

57	Rab GDP dissociation inhibitor beta	P50395	3	0.0336	−1.5
	26S proteasome non-ATPase regulatory subunit 11	O00231	2		

58	Rab GDP dissociation inhibitor beta	P50395	13	0.0000838	−1.5
	Alpha-enolase	P06733	6		
	Adenylosuccinate synthetase isozyme 2	P30520	5		
	Beta-centractin	P42025	3		

59	Tubulin alpha-1C chain	Q9BQE3	5	0.0393	1.5
	Tubulin alpha-1A chain	Q71U36	5		
	Tubulin alpha-4A chain	P68366	4		

60	Alpha-enolase	P06733	7	0.0459	1.5
	Phosphoglycerate kinase 1	P00558	8		

61	Lymphocyte-specific protein 1	P33241	2	0.00972	−1.5
	Secernin-1	Q12765	3		

62	Eukaryotic translation initiation factor 3 subunit H	O15372	3	0.00335	−1.5
	T-complex protein 1 subunit eta	Q99832	4		

63	L-Lactate dehydrogenase B chain	P07195	8	0.00156	−1.5
	Pyruvate kinase isozymes M1/M2	P14618	5		

64	6-Phosphogluconolactonase	O95336	5	0.0029	−1.5
	Endoplasmic reticulum resident protein 29	P30040	4		

65	Pyruvate kinase isozymes M1/M2	P14618	8	0.000801	−1.7
	Sorting nexin-6	Q9UNH7	2		

66	Proteasome subunit beta type-3	P49720	3	0.00454	−1.5
	Triosephosphate isomerase	P60174	5		

67	Pyruvate kinase isozymes M1/M2	P14618	16	0.0219	−1.5

68	14-3-3 protein zeta/delta	P63104	6	0.0053	−1.6

69	4-trimethylaminobutyraldehyde dehydrogenase	P49189	2	0.0183	−1.4
	Caspase-1	P29466	2		

70	Proteasome subunit beta type-4	P28070	2	0.000337	−1.4

71	Chloride intracellular channel protein 1	O00299	6	0.00012	−1.7
	Tumor protein D5	O43399	2		

72	Major vault protein	Q14764	8	0.0122	−1.7
	Ubiquitin-like modifier-activating enzyme 1	P22314	8		

73	Transitional endoplasmic reticulum ATPase	P55072	13	0.0132	−1.8
	Ras GTPase-activating-like protein IQGAP1	P46940	3		

74	Serum albumin	P02768	10	0.00454	−1.7

75	Heat shock protein beta-1	P04792	2	0.00187	1.5

76	Vinculin	P18206	6	0.00886	−1.5

77	Gelsolin	P06396	7	0.0459	−2.1

78	Actin, cytoplasmic 1; actin, cytoplasmic 2	P60709; P63261	5	0.0221	1.9
	Annexin A1	P04083	3		

79	Tryptophan-tRNA ligase, cytoplasmic	P23381	7	0.00185	−1.6

80	Vinculin	P18206	7	0.0369	−1.7

81	Proteasome activator complex subunit 1	Q06323	7	0.0328	−1.5

82	Cofilin-1	P23528	3	0.000586	−1.7

83	Endoplasmin	P14625	5	0.00491	−2.6

84	Alpha-enolase	P06733	4	0.000166	2.4
	Monoglyceride lipase	Q99685	1		

85	Alpha-actinin-1	P12814	2	0.00777	2.4

86	Heterogeneous nuclear ribonucleoprotein H2	P55795	4	0.000792	−2.3
	Heterogeneous nuclear ribonucleoprotein H	P31943	3		

87	Actin, cytoplasmic 1; actin, cytoplasmic 2	P60709; P63261	3	0.00752	2.3
	Alpha-actinin-1	P12814	2		

88	Delta(3,5)-delta(2,4)-dienoyl-CoA isomerase, mitochondrial	Q13011	3	0.000218	−2.2
	Estradiol 17-beta-dehydrogenase 8	Q92506	2		

89	Heterogeneous nuclear ribonucleoproteins A2/B1	P22626	3	0.000986	−2.2

90	Transitional endoplasmic reticulum ATPase	P55072	5	0.00163	−2.1

91	Aconitate hydratase, mitochondrial	Q99798	7	0.000362	−2.1
	Endoplasmin	P14625	2		

92	Endoplasmin	P14625	7	0.000319	−2.0

93	Heat shock 70 kDa protein 1A/1B	P08107	4	0.00155	−2.0
	Dihydrolipoyllysine-residue acetyltransferase component of pyruvate dehydrogenase complex, mitochondrial	P10515	6		
	Stress-70 protein, mitochondrial	P38646	3		
	Annexin A6	P08133	3		

94	Heat shock protein 75 kDa, mitochondrial	Q12931	3	0.00144	−2.0

95	78 kDa glucose-regulated protein	P11021	17	0.0018	−2.5
	Protein disulfide-isomerase A4	P13667	4		
	Endoplasmin	P14625	2		

96	Endoplasmic reticulum resident protein 29	P30040	6	0.00318	−1.9
	ATP synthase subunit gamma, mitochondrial	P36542	2		

97	Endoplasmin	P14625	7	0.0109	−1.9

98	Tropomyosin alpha-4 chain	P67936	4	0.00755	2.1

99	78 kDa glucose-regulated protein	P11021	7	0.00377	−1.8

100	Endoplasmin	P14625	5	0.0108	−1.8

101	40S ribosomal protein SA	P08865	3	0.00000707	−1.8
	Vimentin	P08670	4		

102	F-actin-capping protein subunit beta	P47756	2	0.000949	−1.8

103	78 kDa glucose-regulated protein	P11021	15	0.00946	−2.1

104	Superoxide dismutase [Cu-Zn]	P00441	1	0.00139	1.7
	ATP synthase subunit alpha, mitochondrial	P25705	1		

105	Actin, cytoplasmic 1; actin, cytoplasmic 2	P60709; P63261	4	0.00138	1.7

106	F-actin-capping protein subunit alpha-1	P52907	2	0.00247	1.7

107	Purine nucleoside phosphorylase	P00491	2	0.00353	−1.7
	Dolichyl-diphosphooligosaccharide-protein glycosyltransferase subunit 1	P04843	2		

108	Stress-70 protein, mitochondrial	P38646	10	0.00224	−1.6
	Heat shock cognate 71 kDa protein	P11142	4		

109	Stress-70 protein, mitochondrial	P38646	2	0.0137	1.7

110	Actin, cytoplasmic 1; actin, cytoplasmic 2	P60709; P63261	2	0.00201	1.7

111	Delta(3,5)-delta(2,4)-dienoyl-CoA isomerase, mitochondrial	Q13011	3	0.00338	−1.7

112	60 kDa heat shock protein, mitochondrial	P10809	6	0.00385	1.7
	Integrin beta-3	P05106	4		
	ATP synthase subunit beta, mitochondrial	P06576	2		

113	Aldehyde dehydrogenase, mitochondrial	P05091	9	0.000949	−1.7

114	Heat shock cognate 71 kDa protein	P11142	8	0.0122	−1.7
	Stress-70 protein, mitochondrial	P38646	4		
	Endoplasmin	P14625	4		

115	T-complex protein 1 subunit beta	P78371	5	0.0215	−1.7

116	Moesin	P26038	4	0.000902	−1.6
	T-complex protein 1 subunit zeta	P78371	2		

117	Macrophage-capping protein	P40121	2	0.0114	−1.6

118	Stress-70 protein, mitochondrial	P38646	13	0.0106	−1.7
	Annexin A6	P08133	3		

119	60 kDa heat shock protein, mitochondrial	P10809	11	0.000211	−1.6
	Heterogeneous nuclear ribonucleoprotein K	P61978	2		

120	Elongation factor Tu, mitochondrial	P49411	4	0.00392	−1.5
	Isocitrate dehydrogenase [NADP] cytoplasmic	O75874	2		

121	Pre-mRNA-processing factor 19	Q9UMS4	2	0.00523	−1.6

122	Prohibitin	P35232	9	0.0121	−1.6
	Annexin A2	P07355	6		

123	78 kDa glucose-regulated protein	P11021	6	0.0216	−1.6
	ATP synthase subunit beta, mitochondrial	P06576	4		
	Integrin alpha-IIb	P08514	2		

124	DnaJ homolog subfamily B member 11	Q9UBS4	2	0.00315	−1.6
	Moesin	P26038	2		

125	Protein disulfide-isomerase	P07237	7	0.0422	−1.6

126	Gelsolin	P06396	3	0.0294	1.5

127	14-3-3 protein zeta/delta	P63104	5	0.0337	1.5
	14-3-3 protein eta	Q04917	2		

128	60 kDa heat shock protein, mitochondrial	P10809	18	0.00252	−1.5

129	Endoplasmin	P14625	4	0.0251	−1.6

130	Synaptosomal-associated protein 23	O00161	3	0.0254	1.5

131	Stress-induced-phosphoprotein 1	P31948	1	0.0329	−1.5

132	Calreticulin	P27797	2	0.00435	−1.5

133	UPF0568 protein C14orf166	Q9Y224	4	0.0208	−1.5

134	60 kDa heat shock protein, mitochondrial	P10809	7	0.000412	1.5
	ATP synthase subunit beta, mitochondrial	P06576	2		
	Integrin beta-3	P05106	2		

135	Alpha-enolase	P06733	4	0.0357	−1.5

136	Myosin-9	P35579	5	0.0106	1.5

137	Myosin regulatory light polypeptide 9	P24844	3	0.0325	1.7
	Protein disulfide-isomerase A6	Q15084	2		

138	Endoplasmic reticulum resident protein 29	P30040	6	0.0104	−1.5

139	Peroxiredoxin-4	Q13162	2	0.0362	−1.5

140	Tyrosine-protein phosphatase nonreceptor type 6	P29350	3	0.0319	−1.5
	Protein disulfide-isomerase A3	P30101	2		

141	Protein disulfide-isomerase A3	P30101	2	0.0125	−1.5

142	PDZ and LIM domain protein 1	O00151	2	0.00306	1.5

143	ATP synthase subunit beta, mitochondrial	P06576	18	0.00266	−1.4
	Protein disulfide-isomerase A6	Q15084	6		
	78 kDa glucose-regulated protein	P11021	4		
	Protein disulfide-isomerase	P07237	2		
	Vimentin	P08670	2		

144	Plastin-2	P13796	15	0.000372	−1.6

145	Actin, cytoplasmic 1; actin, cytoplasmic 2	P60709; P63261	3	0.00905	1.5

146	Actin, cytoplasmic 1; actin, cytoplasmic 2	P60709; P63261	4	0.0156	1.8

147	60 kDa heat shock protein, mitochondrial	P10809	9	0.018	1.5

148	Filamin-A	P21333	4	0.000123	2.6

149	Filamin-A	P21333	3	0.00116	2.6

150	Alpha-actinin-1	P12814	7	0.00104	2.4

151	Ficolin-1	O00602	2	0.000967	−2.4

152	Ezrin	P15311	3	0.0136	−2.4

153	Coronin-1A	P31146	1	0.00915	−2.2

154	Alpha-actinin-1	P12814	4	0.00109	2.2

155	Fermitin family homolog 3	Q86UX7	2	0.000261	2.1

156	Filamin-A	P21333	2	0.000568	2.1

157	Alpha-actinin-1	P12814	5	0.0017	2.0

158	Filamin-A	P21333	2	0.0123	1.9

159	Ficolin-1	O00602	2	0.0221	−1.8

160	Alpha-actinin-1	P12814	5	0.000516	1.6
	Filamin-A	P21333	2		

161	14-3-3 protein zeta/delta	P63104	3	0.022	1.8

162	Prohibitin	P35232	3	0.0414	−1.8

163	Actin, cytoplasmic 1; actin, cytoplasmic 2	P60709; P63261	2	0.0133	1.7

164	Nucleoprotein TPR	P12270	1	0.00952	1.7

165	Actin, cytoplasmic 1; actin, cytoplasmic 2	P60709; P63261	5	0.037	1.7
	Filamin-A	P21333	3		
	Beta-parvin	Q9HBI1	2		

166	Alpha-actinin-1	P12814	3	0.00969	1.7

167	Filamin-A	P21333	2	0.0109	1.7

168	Actin, cytoplasmic 1; actin, cytoplasmic 2	P60709; P63261	2	0.00529	2.8

169	Ezrin	P15311	2	0.0174	−1.7

170	Beta-actin-like protein 2	Q562R1	2	0.0226	1.6

171	Filamin-A	P21333	2	0.00975	1.6

172	Filamin-A	P21333	3	0.0319	1.6

173	Actin, cytoplasmic 1; actin, cytoplasmic 2	P60709; P63261	2	0.0202	1.6

174	Actin, cytoplasmic 1; actin, cytoplasmic 2	P60709; P63261	3	0.0207	1.6

175	Myosin-9	P35579	2	0.0225	1.6

176	Actin, cytoplasmic 1; actin, cytoplasmic 2	P60709; P63261	4	0.000991	2.2

177	Filamin-A	P21333	3	0.0131	1.5

178	Filamin-A	P21333	5	0.031	2.1
	Actin, cytoplasmic 1; actin, cytoplasmic 2	P60709; P63261	3		

**Table 3:** Functional association of the identified protein dataset with KEGG cellular pathways.

Annotation (pathway/process)	XD-score	Fisher-test, *q* value
Pathogenic *Escherichia coli* infection	1.3719	3.10*E* − 05
Proteasome	1.2793	4.20*E* − 04
Glycolysis/gluconeogenesis	0.9755	4.20*E* − 04
Pyruvate metabolism	0.9392	1.50*E* − 02
Antigen processing and presentation	0.9051	5.40*E* − 04

**Table 4:** Functional association of the identified protein dataset with Reactome cellular pathways.

Annotation (pathway/process)	XD-score	Fisher-test, *q* value
Formation of tubulin folding intermediates by CCT Tric	3.7875	2.40*E* − 08
Further platelet releasate	3.1660	5.00*E* − 07
Prefoldin mediated transfer of substrate to CCT Tric	2.8529	2.00*E* − 07
Activation of chaperones by IRE1 alpha	2.4375	1.40*E* − 02
Postchaperonin tubulin folding pathway	2.3845	9.60*E* − 04
Chaperonin mediated protein folding	1.6524	2.20*E* − 06
Formation of ATP by chemiosmotic coupling	1.5375	3.80*E* − 02
Smooth muscle contraction	1.4518	1.30*E* − 02
Cell-extracellular matrix interactions	1.4250	4.50*E* − 02
Glycolysis	1.3738	1.40*E* − 02
p53 independent DNA damage response	1.2026	9.60*E* − 04
Stabilization of p53	1.1070	1.30*E* − 03
Regulation of ornithine decarboxylase	1.0779	1.30*E* − 03
VIF mediated degradation of APOBEC3G	1.0779	1.30*E* − 03
Platelet degranulation	1.0545	5.20*E* − 06
SCF beta TRCP mediated degradation of EMI1	1.0500	1.40*E* − 03
Association of Tric CCT with target proteins during biosynthesis	1.0232	3.00*E* − 02

**Table 5:** List of the expression profile groups (A, B, and C) found in the cytosolic subproteome (F1).

Group F1A		
Spot	Protein	Uniprot AC

1	Vinculin	P18206
17	Vinculin	P18206
43	Talin-1	Q9Y490
48	Vinculin	P18206
59	Tubulin alpha-1C chain	Q9BQE3
	Tubulin alpha-1A chain	Q71U36
	Tubulin alpha-4A chain	P68366
26	Proteasome activator complex subunit 1	Q06323
	Coagulation factor XIII A chain	P00488
47	Tubulin beta-1 chain	Q9H4B7

Group F1B		
Spot	Protein	Uniprot AC

56	Actin, cytoplasmic 1; actin, cytoplasmic 2	P60709; P63261
46	Actin, cytoplasmic 1; actin, cytoplasmic 2	P60709; P63261
2	Tubulin alpha-1C chain; tubulin alpha-1B chain	Q9BQE3; P68363
	Annexin A6	P08133
9	Actin, cytoplasmic 1; actin, cytoplasmic 2	P60709; P63261
16	Actin, cytoplasmic 1; actin, cytoplasmic 2	P60709; P63261
	T-complex protein 1 subunit beta	P78371
38	Talin-1	Q9Y490

Group F1C		
Spot	Protein	Uniprot AC

51	Annexin A6	P08133
	Heat shock 70 kDa protein 1A/1B	P08107
	Heat shock 70 kDa protein 1-like	P34931
	Heat shock protein HSP 90-alpha	P07900
7	Leukotriene A-4 hydrolase	P09960
15	Annexin A7	P20073
	Adenosylhomocysteinase	P23526
	Proliferation-associated protein 2G4	Q9UQ80
58	Rab GDP dissociation inhibitor beta	P50395
	Alpha-enolase	P06733
	Adenylosuccinate synthetase isozyme 2	P30520
	Beta-centractin	P42025
36	Proliferation-associated protein 2G4	Q9UQ80
	Annexin A1	P04083
14	Alpha-enolase	P06733
	Adenylosuccinate synthetase isozyme 2	P30520
	Rab GDP dissociation inhibitor beta	P50395
	C-terminal-binding protein 1	Q13363
21	Alpha-enolase	P06733
3	Alpha-enolase	P06733
35	m7GpppX diphosphatase	Q96C86
	Transaldolase	P37837
34	26S proteasome non-ATPase regulatory subunit 14	O00487
23	Proteasome subunit alpha type-6	P60900
	UPF0568 protein C14orf166	Q9Y224
	Hypoxanthine-guanine phosphoribosyltransferase	P00492
18	Triosephosphate isomerase	P60174
66	Proteasome subunit beta type-3	P49720
	Triosephosphate isomerase	P60174
54	Triosephosphate isomerase	P60174
70	Proteasome subunit beta type-4	P28070
27	Heat shock cognate 71 kDa protein	P11142
	Rho-related GTP-binding protein RhoC	P08134
50	Proteasome activator complex subunit 2	Q9UL46

**Table 6:** List of the expression profile groups (A, B, C, D, and E) found in the membrane and membrane organelle subproteome (F2).

Group F2A		
Spot	Protein	Uniprot AC

85	Alpha-actinin-1	P12814
112	60 kDa heat shock protein, mitochondrial	P10809
	Integrin beta-3	P05106
	ATP synthase subunit beta, mitochondrial	P06576
130	Synaptosomal-associated protein 23	O00161
107	Purine nucleoside phosphorylase	P00491
	Dolichyl-diphosphooligosaccharide-protein glycosyltransferase subunit 1	P04843
142	PDZ and LIM domain protein 1	O00151
136	Myosin-9	P35579

Group F2B		
Spot	Protein	Uniprot AC

91	Aconitate hydratase, mitochondrial	Q99798
	Endoplasmin	P14625
94	Heat shock protein 75 kDa, mitochondrial	Q12931
116	Moesin	P26038
	T-complex protein 1 subunit zeta	P78371
132	Calreticulin	P27797
86	Heterogeneous nuclear ribonucleoprotein H2	P55795
	Heterogeneous nuclear ribonucleoprotein H	P31943
83	Endoplasmin	P14625
101	40S ribosomal protein SA	P08865
	Vimentin	P08670

Group F2C		
Spot	Protein	Uniprot AC

122	Prohibitin	P35232
	Annexin A2	P07355
96	Endoplasmic reticulum resident protein 29	P30040
	ATP synthase subunit gamma, mitochondrial	P36542
111	Delta(3,5)-delta(2,4)-dienoyl-CoA isomerase, mitochondrial	Q13011
89	Heterogeneous nuclear ribonucleoproteins A2/B1	P22626
124	DnaJ homolog subfamily B member 11	Q9UBS4
	Moesin	P26038
113	Aldehyde dehydrogenase, mitochondrial	P05091
119	60 kDa heat shock protein, mitochondrial	P10809
	Heterogeneous nuclear ribonucleoprotein K	P61978

Group F2D		
Spot	Protein	Uniprot AC

99	78 kDa glucose-regulated protein	P11021
103	78 kDa glucose-regulated protein	P11021
95	78 kDa glucose-regulated protein	P11021
	Protein disulfide-isomerase A4	P13667
	Endoplasmin	P14625
143	ATP synthase subunit beta, mitochondrial	P06576
	Protein disulfide-isomerase A6	Q15084
	78 kDa glucose-regulated protein	P11021
	Protein disulfide-isomerase	P07237
	Vimentin	P08670
144	Plastin-2	P13796
128	60 kDa heat shock protein, mitochondrial	P10809
114	Heat shock cognate 71 kDa protein	P11142
	Stress-70 protein, mitochondrial	P38646
	Endoplasmin	P14625
118	Stress-70 protein, mitochondrial	P38646
	Annexin A6	P08133
140	Tyrosine-protein phosphatase nonreceptor type 6	P29350
	Protein disulfide-isomerase A3	P30101
88	Delta(3,5)-Delta(2,4)-dienoyl-CoA isomerase, mitochondrial	Q13011
	Estradiol 17-beta-dehydrogenase 8	Q92506

Group F2E		
Spot	Protein	Uniprot AC

97	Endoplasmin	P14625
92	Endoplasmin	P14625
90	Transitional endoplasmic reticulum ATPase	P55072
115	T-complex protein 1 subunit beta	P78371
117	Macrophage-capping protein	P40121
102	F-actin-capping protein subunit beta	P47756

**Table 7:** List of the expression profile groups (A, B) found in the nuclear subproteome (F3).

Group F3A		
Spot	Protein	Uniprot AC
167	Filamin-A	P21333
156	Filamin-A	P21333
176	Actin, cytoplasmic 1; actin, cytoplasmic 2	P60709; P63261
172	Filamin-A	P21333
155	Fermitin family homolog 3	Q86UX7

Group F3B		
Spot	Protein	Uniprot AC

161	14-3-3 protein zeta/delta	P63104
148	Filamin-A	P21333
154	Alpha-actinin-1	P12814
157	Alpha-actinin-1	P12814
150	Alpha-actinin-1	P12814
